# Remote Assessment of Ataxia Severity in SCA3 Across Multiple Centers and Time Points

**DOI:** 10.1002/acn3.70316

**Published:** 2026-01-22

**Authors:** Marcus Grobe‐Einsler, Vivian Maas, Arian Taheri Amin, Jennifer Faber, Tamara Schaprian, Katharina Hill, Matthis Synofzik, Lisa H. Graf, Heike Jacobi, Manuela Lima, Ana F. Ferreira, Bart P. van de Warrenburg, Ilse H. J. Willemse, Dagmar Timmann, Andreas Thieme, Paola Giunti, Hector Garcia‐Moreno, Magda M. Santana, Jeannette Hübener‐Schmid, Elin H. Davies, Thomas Klockgether

**Affiliations:** ^1^ German Center for Neurodgenerative Diseases (DZNE) Bonn Germany; ^2^ Center for Neurology, Department of Parkinson's Disease, Sleep and Movement Disorders, University Hospital Bonn Bonn Germany; ^3^ Department of Neurology, Center for Movement Disorders and Neuromodulation, Medical Faculty Heinrich‐Heine University Düsseldorf Germany; ^4^ Department of Radiology, Medical Faculty Heinrich‐Heine University Düsseldorf Germany; ^5^ Center for Neurology and Hertie Institute for Clinical Brain Research University of Tübingen Tübingen Germany; ^6^ German Center of Neurodegenerative Diseases (DZNE) Tübingen Germany; ^7^ Department of Neurology, University Hospital Heidelberg Heidelberg Germany; ^8^ Faculdade de Ciências e Tecnologia Universidade dos Açores Ponta Delgada Portugal; ^9^ UMIB—Unit for Multidisciplinary Research in Biomedicine, ICBAS—School of Medicine and Biomedical Sciences, University of Porto Porto Portugal; ^10^ Radboud University Medical Center Department of Neurology Nijmegen the Netherlands; ^11^ Department of Neurology and Center for Translational Neuro‐ and Behavioral Sciences (C‐TNBS), Essen University Hospital, University of Duisburg‐Essen Essen Germany; ^12^ Ataxia Centre, Clinical and Movement Neurosciences, UCL Queen Square Institute of Neurology University College London London UK; ^13^ Center for Neuroscience and Cell Biology University of Coimbra (CNC‐UC) Coimbra Portugal; ^14^ Center for Innovative in Biomedicine and Biotechnology (CIBB), University of Coimbra Coimbra Portugal; ^15^ Gene Therapy Center of Excellence (GeneT) Coimbra Portugal; ^16^ Institute for Medical Genetics and Applied Genomics University of Tuebingen Tuebingen Germany; ^17^ Aparito ‐ a Wholly Owned Subsidiary of Eli Lilly and Company Wrexham UK

**Keywords:** ataxia, fluctuation, remote, SARAhome, SCA

## Abstract

**Objective:**

Spinocerebellar ataxia type 3 (SCA3) is a genetically defined ataxia. The Scale for Assessment and Rating of Ataxia (SARA) is a clinician‐reported outcome that measures ataxia severity at a single time point. In its standard application, SARA fails to capture short‐term fluctuations, limiting its sensitivity in trials. To overcome this, we employed SARA^home^, a video‐based, self‐administered tool for high‐frequency, remote ataxia assessment.

**Methods:**

We assessed feasibility and validity of SARA^home^ in 65 SCA3 patients from seven centers. Participants recorded SARA^home^ twice daily for 14 days using a mobile e‐health app. We analyzed adherence, intraindividual fluctuations and their predictors, and evaluated sensitivity to change in a longitudinal substudy of 11 patients.

**Results:**

Adherence to the study protocol was generally high (80.2%) with valid scores in 79.2% of 1459 recordings. Maximum adherence occurred over a 4‐day period (84.8%). Fluctuations ranged 3.0 points between lowest and highest scores (IQR: 2.5–4.5) and 1.0 point based on score IQRs (IQR: 0.5–1.5), corresponding to 10.7% and 3.6% of the maximal SARA^home^ score. Fluctuations showed rough agreement with patient global impression. Greater disease severity and longer CAG repeats were associated with smaller relative fluctuations. Over a median follow‐up of 411 days, SARA^home^ showed higher sensitivity to change than conventional SARA (SRM: 0.67 vs. 0.37).

**Interpretation:**

SARA^home^ is a feasible, innovative video‐based tool for remote, high‐frequency monitoring of ataxia severity. A 4‐day recording effectively captures relevant fluctuations and enhances sensitivity to change, supporting its use in future SCA3 trials.

## 1 Introduction

Spinocerebellar ataxia type 3 (SCA3) is one of the most common autosomal dominantly inherited ataxias worldwide. SCA3 takes a progressive course and leads to increasing disability and premature death. It is caused by unstable expansions of polyglutamine encoding CAG repeats within the *ATXN3* gene, resulting in the formation of abnormally elongated, misfolded ataxin‐3 protein [1]. Targeted therapies for SCA3 are being developed, and first safety trials of antisense oligonucleotides (ASOs) have been initiated (https://clinicaltrials.gov, NCT05160558, NCT05822908).

The clinical hallmark of SCA3 is progressive ataxia. To assess the severity of ataxia, the Scale for Assessment and Rating of Ataxia (SARA) is widely used. SARA is a clinician‐reported outcome measure (ClinRO) that consists of eight items. The SARA sum score ranges from 0 to 40 with higher scores representing more severe ataxia [2]. However, a SARA score taken by a trained rater during a study visit may not be representative of the patient's true condition, as it does not capture the natural fluctuations of ataxia severity that almost all patients experience [3].

To adequately record the extent of fluctuations, repeated assessments made at short intervals, preferably at home, are needed. SARA^home^ is a validated, video‐based instrument that is derived from the original SARA scale and is self‐applied by patients at home. It consists of five SARA items (gait, stance, speech, nose‐finger test, and fast alternating hand movements) and has a maximum score of 28 points. The feasibility of the SARA^home^ application has been demonstrated in a 14‐day pilot study in 12 ataxia patients, which revealed intraindividual differences between the lowest and highest SARA^home^ scores that ranged from 1 to 5.5 [3]. SARA^home^ has since been implemented in an e‐health app to facilitate application in larger clinical trials.

We have used SARA^home^ to remotely monitor ataxia severity in SCA3 patients from the multicentric ESMI cohort [4]. Aims of this study were (1) to demonstrate feasibility of remote monitoring using the SARA^home^ in a multicentre, trial‐like setting, and to explore the potential benefits of remote monitoring in this context, (2) to define the extent of fluctuations of ataxia severity in SCA3, (3) to identify factors that determine the extent of fluctuations in SCA3, and (4) to define the responsiveness of SARA^home^ compared to conventional SARA in SCA3.

## 2 Methods

### 2.1 Patients and Ethics

Patients were consecutively recruited from seven study centers of the European Spinocerebellar Ataxia Type 3/Machado‐Joseph‐Disease Initiative (ESMI) in Germany (Bonn, Essen, Heidelberg, Tuebingen), United Kingdom (London), The Netherlands (Nijmegen), and Portugal (Azores). Inclusion criteria were: (1) genetically confirmed diagnosis of SCA3, (2) clinical manifest ataxia, defined as SARA ≥ 3 at the time of inclusion or during a previous study visit [5], (3) stable wifi connection at home, and (4) the ability to rebuild the SARA^home^ set‐up at home including a 5 m barrier‐free walkway. The study was approved by the local ethics committees of all participating sites and conducted in accordance with the Declaration of Helsinki. All patients provided written informed consent prior to participation.

### 2.2 Study Design and App Assessments

All patients participated in an on‐site baseline visit, during which the SARA and the Montreal Cognitive Assessment (MoCA) tests were performed. For the subsequent remote study phase, patients were instructed and received an iPad and a tripod. To facilitate remote assessment, SARA^home^ was implemented in an e‐health app (ATOM5 by Aparito) that includes safety and set‐up information videos and instruction videos on the correct performance of each SARA^home^ item. Exact SARA^home^ instructions were reported previously [3]. The assessment consists of five examinations (Figure S1). For the gait task, patients start walking from standing directly in front of the camera towards the 5 m point, turn around, and return to the starting point. The tandem gait is performed on a straight line from the 5 m point towards the camera. The fast alternating hand movements (right, then left) and speech recordings are performed while sitting on a chair on the 2 m mark facing the camera. In the nose‐finger test, the chair is rotated 90° to face the wall and patients alternately touch their nose and the mark on the wall at least 5 times (right, then left). All instruction videos remained available to patients via the app for later review. During the on‐site instruction, patients recorded one SARA^home^ assessment under the supervision of an investigator to offer support and corrections, if necessary, and to prevent consecutive errors during home performance.

**FIGURE 1:**
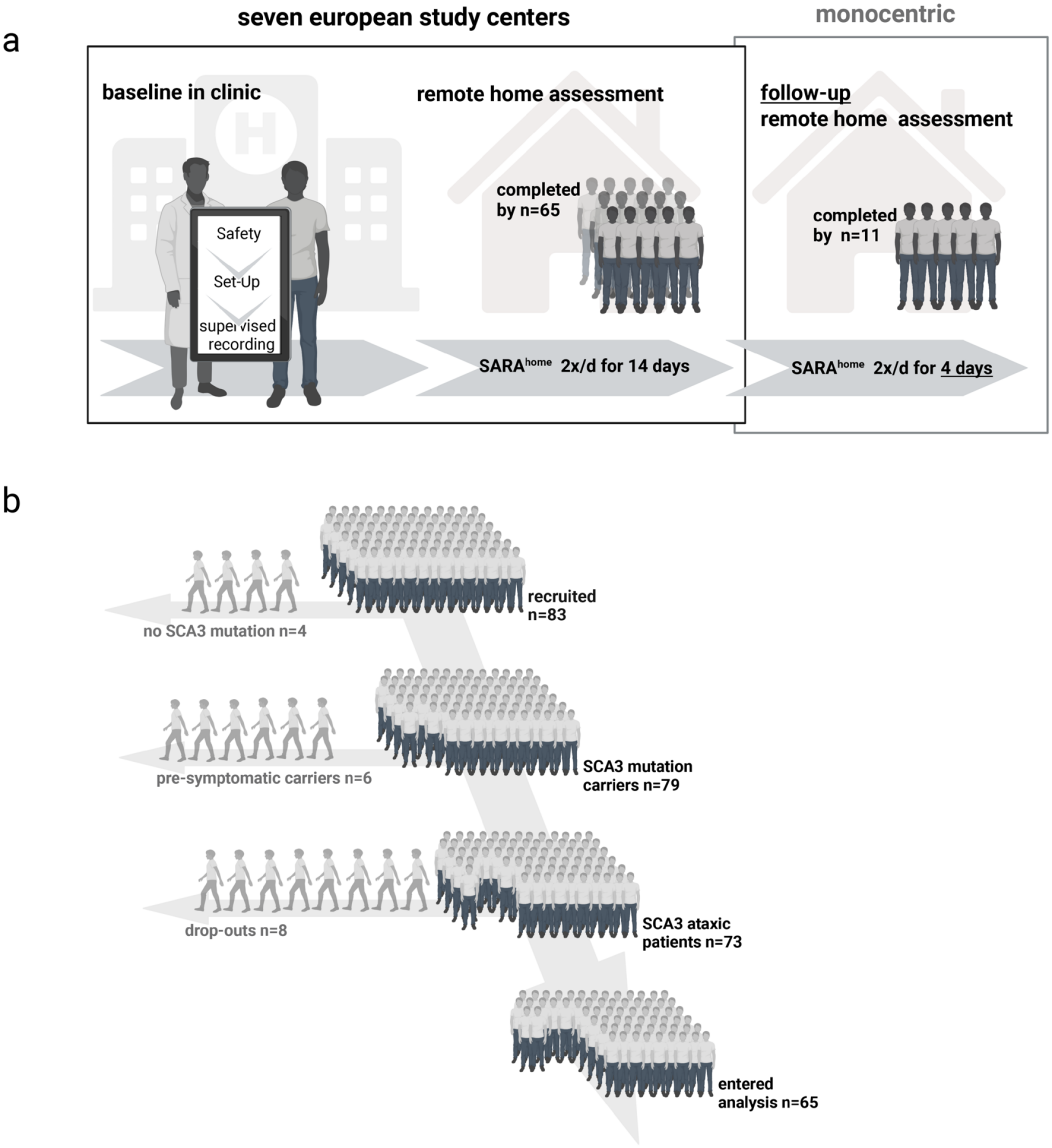
(a) Study design. A baseline study visit included instruction on use of the app, instruction videos on safety and set‐up, and one supervised SARA^home^ recording. During the remote study phase, patients recorded SARA^home^ independently at home twice daily on 14 consecutive days. Prior to every recording, patients reported their subjective severity of ataxia at the time of recording, along with possible influencing factors. (b) Recruitment flow chart.

The on‐site visit was followed by a remote home‐phase, during which patients independently recorded SARA^home^ twice daily for 14 consecutive days (Figure 1). The app sends out push messages as a reminder for upcoming assessments. Videos were recorded and submitted directly via the app. Before each SARA^home^ recording, patients were asked for their subjective assessment of ataxia severity at the time of recording. The reported patient global impression (PGI) was selected from seven categories (very much worse, much worse, minimally worse, no change, minimally improved, much improved, very much improved). Reports of worsening or improvements triggered a second question on possible reasons.

A subset of participants from the study center in Bonn underwent a follow‐up assessment during the next consecutive ESMI study visit after 1 year, including an onsite evaluation followed by SARA^home^ twice daily for four consecutive days (see study design in Figure 1).

### 2.3 Centralized Rating

Centralized assessment of the videos, performance quality, and ratings was performed by five SARA‐certified investigators [6]. Reasons to fail quality control included sections that did not capture the complete assessment or severe performance errors that disqualified for a valid rating (e.g., an insufficient number of repetitions in the fast alternating hand movements, or wearing shoes during stance performance). To reduce inter‐rater variability, consecutive videos from one patient were predominantly rated by a single rater. The Dutch and Portuguese speech recordings were rated by one additional investigator from the respective study site.

### 2.4 Statistical Analysis

For the statistical analysis, patients with fewer than four submitted videos throughout the recording period of 14 days were treated as dropouts. To address the influence of daytime and training (consecutive days) on SARA^home^ sum scores, we calculated two‐way ANOVAs. To determine how many days of recordings are required to obtain a representative estimate of the entire 14‐day period, we estimated mean values and 95% confidence intervals (95% CI) of SARA^home^ sum scores for cumulative days.

We used two measures of the extent of fluctuations: (1) Fluct_range_ defined as the difference between the lowest and highest individual scores, and (2) Fluct_IQR_ defined as the interquartile range (IQR) of the individual scores.

We used gamma generalized linear models with a log link and backward selection to predict adherence rates and Fluct_IQR_ based on available clinical data from the baseline visits. The model to predict the fluctuations was weighted by the number of SARA^home^ sum scores contributed by a patient to consider the adherence when fitting the data. To analyze the association between PGI and objective changes in disease severity, we calculated the deviation of the corresponding SARA^home^ score from the median of all measurements of the respective participant during the recording period for each PGI category. Progression of SARA and SARA^home^ over time and sensitivity to change were determined using one‐tailed paired samples *t*‐tests and the standardized response mean (SRM). The assumptions underlying the statistical methods, including linearity, homoscedasticity, normality of residuals, independence of observations for the linear regression model, and homogeneity of variances, were tested and confirmed to be met, ensuring the validity of the analysis. All analyses were performed using R Software for Statistical Computing, version 4.3.1 (R Core Team 2022: R: A Language and Environment for Statistical Computing, R Foundation for Statistical Computing, Vienna, Austria).

## 3 Results

### 3.1 Enrollment

Between September 2021 and January 2024, we enrolled 83 participants (10 from the Azores, 20 from Bonn, 6 from Essen, 3 from Heidelberg, 20 from London, 10 from Nijmengen and 14 from Tübingen). Of these, 73 had a genetically confirmed diagnosis of SCA3 and a SARA score ≥ 3. Eight patients dropped out after inclusion. Ultimately, 65 SCA3 patients were included in the cross‐sectional analysis and recorded SARA^home^ via an e‐health app twice daily for 14 days, following a baseline assessment that included detailed methodological instructions. Eleven SCA3 patients from the study centre Bonn completed a follow‐up visit that included a conventional SARA followed by four consecutive days of SARA^home^ recording twice daily at a median interval of 411 days (IQR 366–547). The study design and recruitment flowchart are illustrated in Figure 1, baseline characteristics of the study participants are provided in Table 1 and Figure S2.

**TABLE 1:** Patient characteristics.

	All	Follow‐up cohort
Age, Mean ± SD, [*n*]	51.5 *±* 10.7, [65]	50.1 *±* 10.3, [11]
Sex, m/f, [*n*]	35/30, [65]	5/6, [11]
Repeat length (expanded), Mean ± SD, [*n*]	68 *±* 4.5, [63]	66.7 *±* 6.8, [10]
SARA, Median (IQR), [*n*]	9.5 (5.5–13), [65]	10.5 (5.5–12.5), [11]
Gait, Median (IQR), [*n*]	3 (2–3), [65]	3 (2–3), [11]
Stance, Median (IQR), [*n*]	2 (0–2), [65]	2 (0–2), [11]
Sitting, Median (IQR), [*n*]	0 (0–1), [65]	0 (0–1), [11]
Speech, Median (IQR), [*n*]	1 (0–2), [65]	1 (0–2), [11]
Finger chase, Median (IQR), [*n*]	1 (0.5–1), [65]	1 (0.5–1), [11]
Alternating hands, Median (IQR), [*n*]	1 (0.5–1.5), [65]	1 (0.5–1.5), [11]
Nose finger, Median (IQR), [*n*]	1 (1–1), [65]	1 (1–1), [11]
Heel shin slide, Median (IQR), [*n*]	1.5 (1.5–2), [65]	1.5 (1.5–2), [11]
MoCA, Median (IQR), [*n*]	27 (25–29), [55]	27 (26–29), [11]

### 3.2 Adherence

**FIGURE 2:**
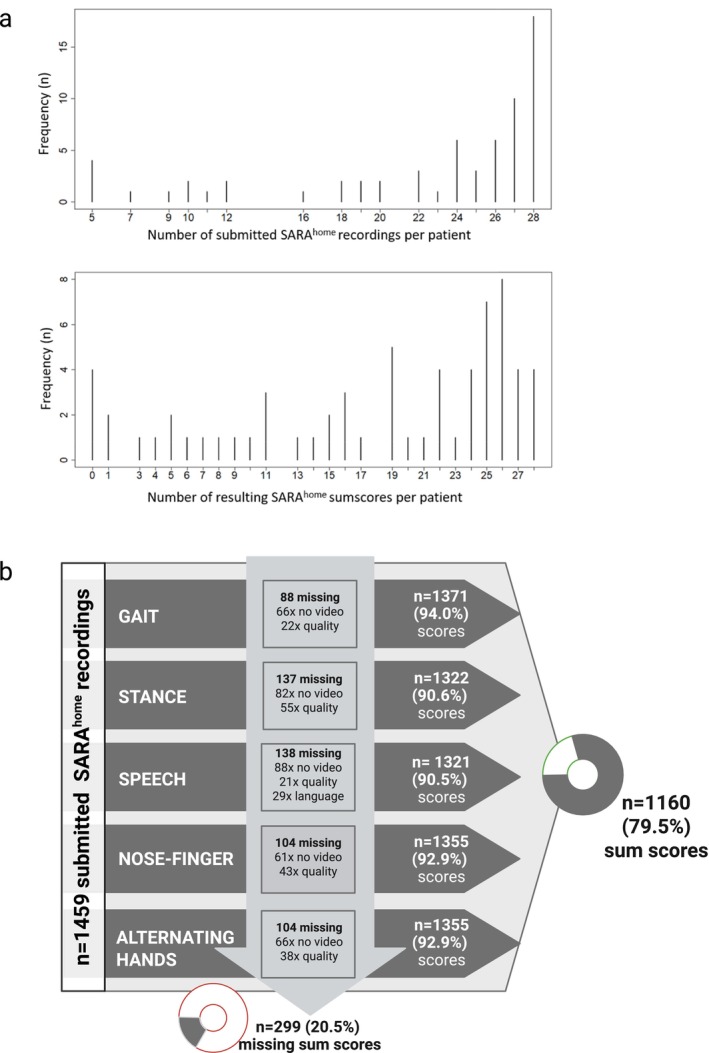
Descriptive statistics of adherence to study protocol and ratings. (a) Frequency distribution diagrams of the number of submitted SARA^home^ recordings per patient (top) and the derived SARA^home^ scores per patient (bottom). (b) Flow chart diagram of recorded videos and derived scores. Sixty‐five patients recorded SARA^home^ videos on 1459 occasions that were subject to centralized quality control and rating. The number of missing videos, derived scores, and reasons for missing differed between the items. The scored item recordings resulted in 1160 SARA^home^ sum scores.

Study participants completed a median number of 26 (IQR 20–25) SARA^home^ recordings, adding up to 1459 recordings. Figure 2 displays the frequency distribution of recordings and descriptive statistics of derived SARA^home^ scores. The adherence rates from cumulative days rose from 75.4% on day 1 to 84.8% after 4 days and then gradually decreased to an overall adherence of 80.2% after 14 days. Forty‐seven patients (72.3%) completed > 20 recordings (adherence: > 71.4%), whereas 34 patients (52.0%) completed > 25 recordings (adherence: > 92.9%). From the 1459 SARA^home^ recordings, raters were able to obtain 1371 (94.0%) valid gait, 1322 (90.6%) stance, 1321 (90.5%) speech, 1355 (92.9%) alternating hand movements, and 1355 (92.9%) nose‐finger scores. Reasons for the raters not being able to score a SARA^home^ item included incomplete video uploads, incorrect performance, and poor recording quality. A total of 1160 SARA^home^ sum scores (median 21 with IQR 11–25 scores per patient) could be derived from the 1459 recordings (79.2%). Descriptive statistics of derived SARA^home^ scores are provided in Table S1.

To predict the adherence based on baseline data, we used a multivariate model. Of the included predictors (age, sex, expanded CAG repeat length, SARA, and MoCA), only age reached the level of significance (β = 0.019895, SE = 0.006585, *t* = 3.021, *p* = 0.00432), indicating that higher age was a predictor of better adherence. This model reduced unexplained deviance compared to a null model by 16.0% (Table S2).

### 3.3 Daytime and Training Effect

To investigate whether the time of recording (morning vs. evening) or training (consecutive days of recording) influences SARA^home^ scores, we performed a two‐way ANOVA. This analysis showed no significant interaction (F (13, 1132) = 0.145, *p* = 1.000), or simple main effects for the two predictors, neither for the SARA^home^ sum scores (F (1, 1132) = 0.057, *p* = 0.812 for time of recording; F (13, 1132) = 0.430, *p* = 0.959 for training), nor for any single item (Table S3).

### 3.4 Reliability of SARA^home^ Recordings

**FIGURE 3:**
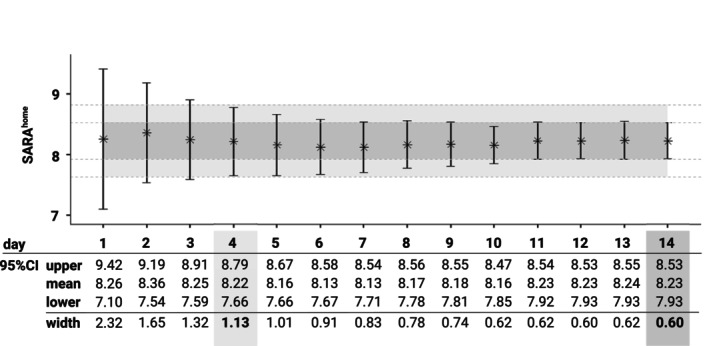
95% confidence intervals (95% CI) of SARA^home^ scores depending on the cumulated number of recording days. Mean values are indicated by stars. The dark gray area indicates the 95% CI of 14 days of recordings. Starting from day 4, the 95% CIs were less than double of the 14‐day 95% CI (light gray area).

To obtain a measure of the reliability of SARA^home^ scores as a function of the duration of the recording, we determined the width of 95% CIs of the SARA^home^ sum scores for cumulative days. The width of 95% CIs dropped from 2.32 for 1‐day to 0.6 for 14‐day recordings. For all recording periods longer than 3 days, 95% CIs intervals were less than twice the 14‐day value (Figure 3).

### 3.5 Relation to Patient‐Reported Ataxia Severity

During the 1459 SARA^home^ recordings, study participants rated their ataxia severity as unchanged in 988, improved in 103 (minimally improved: 96, much improved: 5, very much improved: 2), and worsened in 322 instances (minimally worse: 248, much worse: 71, very much worse: 3). In instances in which participants rated severity as much improved, the SARA^home^ decreased by 0.5 compared to the individual median score, while it increased by 0.25 and 0.5 in those who rated severity as much worse and very much worse, respectively. For all other PGI categories, SARA^home^ did not deviate from the median (Table 3). Reported reasons for subjective improvement included restedness and good mood, whereas those for worsening included tiredness, exhaustion, and physical therapy/training (Figure S3).

### 3.6 Extent of Fluctuations

To measure the extent of fluctuations, we used Fluct_range_ defined as the difference between the lowest and highest individual scores, and Fluct_IQR_, a more conservative measure defined as the interquartile range (IQR) of the individual scores (summarized in Table 2). The median Fluct_range_ was 3.0 (IQR: 2.5–4.5). As the SARA^home^ items have different scoring ranges (0–8 points for gait, 0–6 points for stance and speech, 0–4 points for alternating hand movements and nose‐finger), we converted the differences between the lowest and highest individual item scores to a normalized scale. The normalized gait item scores showed the smallest fluctuations (0.23), followed by the nose‐finger (0.24), the speech (0.27), the stance (0.31), and the fast alternating hand movements items (0.38). The median Fluct_IQR_ was 1.0 (IQR 0.5–1.5). The corresponding normalized item fluctuations (Fluct_IQR_) were 0.0 for gait, stance, speech, and nose‐finger, and 0.1 for alternating hands.

**TABLE 2:** Fluctuation of SARA^home^ sum score and single items provided as the difference between the lowest and highest individual scores (Fluct_range_), and as the interquartile range (IQR) of the individual scores (Fluct_IQR_). Item scores are additionally provided as normalized values.

Score	Fluct_range_		Fluct_IQR_	
	Median (IQR)	Normalized	Median (IQR)	Normalized
SARA^home^	3.0 (2.5–4.5)	na	1.0 (0.5–1.5)	na
Gait	1.0 (1.0–2.0)	0.23	0.0 (0.0–1.0)	0.00
Stance	1.0 (0.0–2.0)	0.31	0.0 (0.0–0.1)	0.00
Speech	1.0 (1.0–1.0)	0.27	0.0 (0.0–1.0)	0.00
Nose‐finger	1.0 (0.5–1.0)	0.24	0.1 (0.0–0.5)	0.00
Alternating hands	1.0 (1.0–0.5)	0.38	0.5 (0.0–0.5)	0.10

**TABLE 3:** Deviation of the corresponding SARA^home^ scores from the median of all measurements of the respective participant during the recording period for each patient global impression (PGI) category.

PGI	Median	IQR	*n*
Very much improved	0.00	0.00–0.00	2
Much improved	−0.50	−0.75–0.00	5
Minimally improved	0.00	−0.50–1.00	83
No change	0.00	−0.50–0.50	780
Minimally worse	0.00	−0.25–0.50	201
Much worse	0.25	−1.13–1.00	59
Very much worse	0.50	0.50–7.13	3

### 3.7 Predictors of Fluctuations

To predict the extent of fluctuations relative to the individual ataxia severity, we used a multivariate model with the quotient of Fluct_IQR_ and individual median SARA^home^ score as the dependent variable. Following backward stepwise selection, the final model maintained the predictors age, expanded CAG repeat length, SARA score, MoCA score, and the number of recordings per patient, but omitted sex. The model reduced unexplained deviance by 33% compared to a null‐model. Baseline SARA score (*β* = −0.05, SE = 0.02, *t* = 2.20, *p* = 0.03) and expanded CAG repeat length (*β* = −0.08, SE = 0.03, *t* = −2.42, *p* = 0.02) were significant negative predictors of the extent of fluctuation. Specifically, the normalized SARA^home^ IQR decreased by 5% with each additional baseline SARA scoring point, and by 8% with each additional expanded CAG repeat (Table S2). Comparison of SARA^home^ fluctuations among patient with (MoCA ≤ 25) and without (MoCA > 25) cognitive impairments using Wilcoxon Rank‐Sum Tests, showed no significant difference between the groups for total scores and all items except gait (*p* = 0.05, Cohen's *r* 0.28) (Table S4).

### 3.8 Responsiveness

Eleven SCA3 patients from the study site Bonn (Germany) performed a follow‐up on‐site study visit in conjunction with a consecutive 4‐day SARA^home^ recording after a median interval of 411 days (IQR 366–547) (see study design in Figure 1). The conventional SARA scores did not significantly increase between the first and second recording period (9.9 ± 4.9 to 10.4 ± 5.3, *p* = 0.25; SRM = 0.37). In contrast, the mean SARA^home^ score derived from the 4‐day recording increased from 6.9 ± 3.2 to 8.1 ± 2.91 (*p* = 0.03) with an SRM of 0.67 (Table S5).

## 4 Discussion

SARA^home^ is the only validated video‐based, self‐applied instrument that allows the repeated remote assessment of ataxia severity at home. This allows for seamless integration of the assessment into everyday life and enables assessments to be conducted under ecologically valid, everyday conditions. We have used SARA^home^ in SCA3 patients of the ESMI cohort and observed an adherence larger than 80% demonstrating the feasibility of the SARA^home^ application in a multicenter, trial‐like setting. Fluctuations—defined as the difference between the lowest and highest individual scores—were substantial in SCA3 patients and amounted to more than 10% of the maximal score. Score changes showed a rough agreement with the subjective assessment of ataxia by the participants. In a longitudinal substudy, SARA^home^ was more responsive than the conventional SARA.

A critical question concerns the nature of the observed fluctuations. The rough agreement between SARA^home^ score changes and patients' subjective assessment, and the association of the fluctuations with biological factors, suggests that variability in patients' performance is the cause of the fluctuations. However, we cannot rule out that variability in the ratings may have contributed to the observed fluctuations. As videos from one patient were predominantly rated by a single rater, this potential confounding factor can only arise from intra‐rater variability, whereas inter‐rater variability does not play a role. Notably, previous studies reported Intraclass Correlation Coefficients (ICC) for intra‐rater variability of the conventional SARA as 0.98–0.99, which suggests that intra‐rater variability alone does not explain the extent of the observed fluctuations [7, 8].

Despite a considerable burden to the patients caused by the combination of their physical impairment and the time‐intensive study design with a baseline visit, followed by two daily assessments over 14 consecutive days, we observed an overall adherence of 80.2% with a maximum of 84.8% for the first 4 days of recording. Raters were able to obtain valid item scores from 90.5% to 94.0% of the SARA^home^ recordings. However, a complete SARA^home^ score was obtained only from 79.5% of the recordings. Adherence and analyzability were comparable with those of other digital health assessments, many of which are less burdensome than SARA^home^ [9, 10, 11]. We identified age as a significant, albeit weak predictor of adherence. This is in accordance with adherence to medication, which has been shown to be higher in older than in younger individuals across various diseases [12]. Cognitive impairment did not have a significant effect on fluctuations of disease severity. However, group sizes were too small to draw general assumptions for feasibility in cognitively impaired patients. Furthermore, we may have overestimated feasibility of SARA^home^, as certain conditions required for inclusion (stable WiFi connection, five meters barrier‐free walkway) are not always met in real life. Another limitation of this study is the underrepresentation of patients with very severe ataxia and severe cognitive impairments, which limits generalizability of our results.

Remote home‐based assessment facilitates the inclusion of more severely affected patients, who may have limited access to clinical trial sites. This approach facilitates a more authentic evaluation of patients' functional impairments in their daily lives and thus supports patient‐centered disease management. Whilst continuous sensor‐based monitoring may capture everyday impairments in greater detail, the strong alignment of SARA^home^ with the established SARA ensures comparability with on‐site assessments and data from natural history studies.

Based on the analysis of the width of 95% CIs of the SARA^home^ sum scores, we conclude that a 4‐day recording period allows a reliable estimation of the “true” score and would thus be of sufficient length to adequately capture fluctuations. As discussed above, a 4‐day recording has the added advantage of higher adherence. As measures of the extent of fluctuations, we used Fluct_range_, defined as the difference between the lowest and highest individual scores, and Fluct_IQR_, defined as the interquartile range (IQR) of the individual scores. While Fluct_range_ shows the full range of fluctuation, Fluct_IQR_ is a more conservative but also more robust measure. The latter also provided better model stability compared to Fluct_range_.

Other than our previous pilot study, this study was performed in a large cohort of genetically homogeneous ataxia patients [3]. Nevertheless, the observed extent of fluctuations was similar in both studies. In addition, both studies did not detect an effect of the time of recording or a training effect during the 14 days of recording. We identified baseline SARA and expanded CAG repeat length as negative predictors of the extent of fluctuations relative to individual disease severity. Thus, the extent of relative fluctuations is predicted to be lower in patients with more severe ataxia and larger expanded CAG repeat length, notwithstanding the fact that the absolute extent of fluctuations is larger in more severely affected patients. This apparent discrepancy reflects the different reference frames: relative fluctuation decreases with higher baseline severity because variability constitutes a smaller proportion of the total deficit, whereas absolute fluctuation is greater in advanced disease due to reduced compensatory reserve and higher intrinsic instability.

The results of our study raise critical questions about the design of interventional studies in SCA3. We provide clear evidence that there are relevant short‐term fluctuations of ataxia severity, which is in line with paradoxical improvements of disease severity in individual SCA3 patients in natural history studies [13]. Since all records from one patient were rated by the same examiner (except for the Dutch and Portuguese speech recordings), the variability observed within the 14‐day recording period cannot be explained solely by unfavorable psychometric properties of SARA. They are rather the expression of biologically determined variability of test performance. Future automation of the rating process utilizing motion tracking technology and machine learning will potentially increase reliability and objectivity. The full extent of the SARA^home^ fluctuations was considerably larger than the clinically meaningful change of SARA, which has been specified with 0.7 and 2.2 [14, 15]. Thus, the SARA ratings of single study visits include a random factor of a magnitude that may blur the effect of an intervention. The average of repeated SARA^home^ ratings, on the other hand, will result in a more valid measure of ataxia severity. Our data show that a 4‐day recording is well accepted by patients and sufficient to adequately capture fluctuations. The rationale of this approach is strongly supported by the results of the longitudinal substudy showing that mean SARA^home^ score of a 4‐day recording was more sensitive to detect changes than a conventional SARA score obtained at a study visit. As the longitudinal study was performed in only 11 patients from a single centre, there is an urgent need to perform a larger longitudinal study of the 4‐day SARA^home^ recording.

## Author Contributions

Marcus Grobe‐Einsler: Project Administration, Funding Acquisition, Methodology, Formal Analysis, Investigation, Visualization, Writing (Original Draft Preparation); Thomas Klockgether: Conceptualization, Supervision, Project Administration, Funding Acquisition, Methodology, Writing (Original Draft Preparation); Tamara Schaprian: Methodology, Formal Analysis, Visualization, Writing (Review and Editing); Vivian Maas, Arian Taheri Amin, Jennifer Faber, Katharina Hill, Matthis Synofzik, Lisa H. Graf, Heike Jacobi, Manuela Lima, Ana F. Ferreira, Bart P. van de Warrenburg, Ilse H.J. Willemse, Dagmar Timmann, Andreas Thieme, Paola Giunti, Hector Garcia‐Moreno, Magda M. Santana, Jeannette Hübener‐Schmid: Investigation, Writing (Review and Editing); Elin H. Davies: Software, Data Curation, Writing (Review and Editing).

## Funding

This study was funded by the National Ataxia Foundation (NAF) and Ataxia UK.

## Conflicts of Interest

Marcus Grobe‐Einsler received research support from the German Ministry of Education and Research (BMBF) within the European Joint Program for Rare Diseases (EJP‐RD) 2021 Transnational Call for Rare Disease Research Projects (funding number 01GM2110), and received consulting honoraria from Biogen, all unrelated to this study. Jennifer Faber receives research support from the National Ataxia Foundation (NAF), the Advanced Clinician Scientist Programme (ACCENT, funding code 01EO2107; the ACCENT Program is funded by the German Federal Ministry of Education and Research (BMBF)) and is a fellow of the Hertie Network of Excellence in Clinical Neuroscience. She received consultancy fees from Vico Therapeutics and Biogen, unrelated to the present manuscript. Ana F. Ferreira received honoraria for lectures from the University of the Azores. Matthis Synofzik received consulting honoraria from UCB Pharmaceuticals, Prevail Pharmaceuticals, Ionis Pharmaceuticals, Orphazyme Pharmaceuticals, Servier Pharmaceuticals, Reata Pharmaceuticals, AviadoBio, GenOrph, Biohaven, Zevra, Lilly, and Solaxa, all unrelated to the present manuscript. He and Lisa H. Graf are supported by the European Union, project European Rare Disease Research Alliance (ERDERA), GA no 101156595, funded under call HORIZON‐HLTH‐2023‐DISEASE‐07 (to Matthis Synofzik and Lisa H. Graf). Andreas Thieme held a clinician scientist position that was supported by the DFG in the framework of the DFG Clinician Scientist Programme UMEA (FU 356/12–1 and 12–2) and received travel support from the German Academic Exchange Service (DAAD). Dagmar Timmann received funding from the DFG, EU, MERCUR, and Bernd Fink Foundation unrelated to this work. Magda M. Santana was supported by Fundação para a Ciência e Tecnologia, Portugal (2023.06020.CEECIND/CP2832/CT0010). Bart P. van de Warrenburg received research support from ZonMw (446002002), from the Dutch Scientific Organization, Hersenstichting, the Brugling foundation, and the Christina Foundation and received consulting fees from Biogen, Servier, Biohaven and honoraria from the Malaysia movement disorder society and the Belgian Neurological Society. He is chair of the ataxia study group MDS and Ataxia ACT (AGI) and of the Dutch ataxia guideline, holds licenses in BSL/Springer Nature. Paola Giunti has received grants and honoraria for advisory board from Vico Therapeutics, honoraria for advisory board from Triplet Therapeutics, grants and personal fees from Reata Pharmaceutical, grants from Wave. Paola Giunti and Hector Garcia‐Moreno work at University College London Hospitals/University College London, which receives a proportion of funding from the Department of Health's National Institute for Health Research Biomedical Research Centre's funding scheme. Paola Giunti received funding from CureSCA3 in support of Hector Garcia‐Moreno work. Elin H. Davies is an employee of Aparito and shareholder of Eli Lilly. Thomas Klockgether received research support from the Bundesministerium für Bildung und Forschung. All other authors declare no conflicts of interest. This publication is an outcome of ESMI, an EU Joint Programme—Neurodegenerative Disease Research (JPND) project (see www.jpnd.eu). ESMI is supported through the following funding organizations under the aegis of JPND: Germany, Federal Ministry of Education and Research (BMBF; funding codes 01ED1602A/B); Netherlands, The Netherlands Organization for Health Research and Development; United Kingdom, Medical Research Council (MR/N028767/1).

## Supporting information


**Table S1:** Descriptive statistics of all available remote SARA^home^ assessments at home.
**Table S2:** Summary of multivariate models to predict adherence rates and relative fluctuations of ataxia severity (quotient of Fluct_IQR_ and individual median SARA^home^ score), weighted by the number of SARA^home^ scores, so patients with higher scores are given a higher weight in the model. Significant *p* values are printed bold.
**Table S3:** Summary of two‐way ANOVA results to investigate impact of time of recording (morning vs. evening) or training effects (consecutive days of recording) on ataxia severity. Degrees of Freedom (df): Indicates the number of levels or comparisons for each factor and interaction, Sum of Squares (SS): Represents the variance attributable to each factor or interaction, Mean Square (MS): The variance per degree of freedom, *F*‐statistic: Tests the ratio of explained variance to residual variance, *p* value: Indicates the significance of the effect and *η* ^2^_*p*_ (Eta squared partial): Proportion of variance explained by each factor or interaction, relative to total variance (excluding error).
**Table S4:** Results of Wilcoxon Rank‐Sum Tests Comparing SARA^home^ score ranges between patients with (MoCA ≤ 25) and without (MoCA > 25) cognitive impairments. Med = Median, IQR = Interquartile Range, *p* values < 0.05 are printed bold.
**Table S5:** SARA^home^ and SARA scores at baseline (V1) and during a follow‐up (V2) after a median interval of 411 days (IQR 366–547). SARA^home^ scores were calculated as means from four consecutive days of assessment. Results were compared with *t*‐test.
**Figure S1:** SARA^home^ assessment. SARA^home^ consists of five out of eight SARA items, including examination of gait and stance, speech disturbance, fast alternating hands movements, and the nose‐finger test. Participants require a smart device with camera and 5 m barrier‐free walkway. The examination is video‐recorded using an e‐health app (ATOM5 by Aparito) for centralized rating.
**Figure S2:** Frequency distribution of variables SARA (a), CAG repeat length of the longer allele (b) and MoCa score (c) at baseline.
**Figure S3:** Frequency list of reasons for changes in ataxia severity reported by patients. Red bars (top) show reported reasons for worsenings of ataxia severity (in 322 instances), green bars (bottom) show reasons for improvements (in 103 instances). Relative frequencies are provided in brackets behind the bars.

## Data Availability

The data supporting the results of this study are available on request from the corresponding author and agreement of the ESMI consortium. The data are not publicly available as they contain information that could compromise the privacy of the research participants.
