# Supplementary material for: Remote Assessment of Ataxia Severity in SCA3 Across Multiple Centers and Time Points
Source: Ann Clin Transl Neurol. 2026 Jan 22;13(7):1370–8. doi: 10.1002/acn3.70316 (PMC13358552; doi:10.1002/acn3.70316)
Supplement: Supplementary file 1 — Table S1: Descriptive statistics of all available remote SARAhome assessments at home. Table S2: Summary of multivariate models to predict adherence rates and relative fluctuations of ataxia severity (quotient of FluctIQR and individual median SARAhome score), weighted by the number of SARAhome scores, so patients with higher scores are given a higher weight in the model. Significant p values are printed bold. Table S3: Summary of two‐way ANOVA results to investigate impact of time of recording (morning vs. evening) or training effects (consecutive days of recording) on ataxia severity. Degrees of Freedom (df): Indicates the number of levels or comparisons for each factor and interaction, Sum of Squares (SS): Represents the variance attributable to each factor or interaction, Mean Square (MS): The variance per degree of freedom, F‐statistic: Tests the ratio of explained variance to residual variance, p value: Indicates the significance of the effect and η 2 p (Eta squared partial): Proportion of variance explained by each factor or interaction, relative to total variance (excluding error). Table S4: Results of Wilcoxon Rank‐Sum Tests Comparing SARAhome score ranges between patients with (MoCA ≤ 25) and without (MoCA > 25) cognitive impairments. Med = Median, IQR = Interquartile Range, p values < 0.05 are printed bold. Table S5: SARAhome and SARA scores at baseline (V1) and during a follow‐up (V2) after a median interval of 411 days (IQR 366–547). SARAhome scores were calculated as means from four consecutive days of assessment. Results were compared with t‐test. Figure S1: SARAhome assessment. SARAhome consists of five out of eight SARA items, including examination of gait and stance, speech disturbance, fast alternating hands movements, and the nose‐finger test. Participants require a smart device with camera and 5 m barrier‐free walkway. The examination is video‐recorded using an e‐health app (ATOM5 by Aparito) for centralized rating. Figure S2: Frequency distribu [file ACN3-13-1370-s001.docx]

***Supplementary Table 1:*** *Descriptive statistics of all available remote SARA^home^ assessments at home.*

|  |  | **Main study** | **Follow-up cohort** | |
| --- | --- | --- | --- | --- |
| **Score** |  |  | **Baseline** | **Follow-up** |
| **SARA^home^ sum-score** | *median (IQR)* | 8.0 (5,5-10) | 6.0 (5.0-10.0) | 8.0 (6,5-10) |
| ***Gait*** | *median (IQR)* | 2.0 (2.0-3.0) | 2.0 (2.0-3.0) | 2.0 (2.0-3.0) |
| ***Stance*** | *median (IQR)* | 2.0 (0.0-2.0) | 1.0 (0.0-2.0) | 2.0 (1.0-2.0) |
| ***Alternating Hands*** | *median (IQR)* | 1.0 (0.0-2.0) | 1.0 (1.0-2.0) | 2.0 (1.0-2.0) |
| ***Nose-Finger*** | *median (IQR)* | 1.0 (1.0-1.0) | 1.0 (1.0-1.0) | 1.0 (1.0-2.0) |
| ***Speech*** | *median (IQR)* | 1.5 (1.0-2.0) | 1.0 (0.0-2.0) | 1.0 (0.0-2.0) |

***Supplementary Table 2:*** *Summary of multivariate models to predict adherence rates and relative fluctuations of ataxia severity (quotient of Fluct_IQR_ and individual median SARA^home^ score), weighted by the number of SARA^home^ scores, so patients with higher scores are given a higher weight in the model. Significant p values are printed bold.*

|  | Predictor | Estimate | Std. Error | t-value | p-value |
| --- | --- | --- | --- | --- | --- |
| **Adherence rate** | **(Intercept)** | 0.77 | 1.20 | 0.64 | 0.53 |
|  | **SARA** | -0.00 | 0.01 | -0.29 | 0.77 |
|  | **Age** | 0.02 | 0.01 | 3.02 | **0.004** |
|  | **Sex (male)** | -0.05 | 0.09 | -0.58 | 0.57 |
|  | **MoCA** | -0.01 | 0.01 | -0.50 | 0.62 |
|  | **Repeat length (expanded)** | 0.02 | 0.01 | 1.75 | 0.09 |
|  |  |  |  |  |  |
| **Relative fluctuation** | **(Intercept)** | 4.10 | 3.02 | 1.36 | 0.18 |
|  | **SARA** | -0.05 | 0.022 | -2.20 | **0.03** |
|  | **Age** | -0.03 | 0.02 | -1.39 | 0.17 |
|  | **MoCA** | 0.06 | 0.036 | 1.81 | 0.08 |
|  | **Number of videos** | -0.02 | 0.022 | -0.75 | 0.46 |
|  | **Repeat length (expanded)** | -0.08 | 0.03 | -2.42 | **0.02** |

***Supplementary Table 3:*** *Summary of two-way ANOVA results to investigate impact of* time of recording (morning vs. evening) or training effects (consecutive days of recording) on ataxia severity. ***Degrees of Freedom (df)****: Indicates the number of levels or comparisons for each factor and interaction,* ***Sum of Squares (SS)****: Represents the variance attributable to each factor or interaction,* ***Mean Square (MS)****: The variance per degree of freedom,* ***F-statistic****: Tests the ratio of explained variance to residual variance,* ***p-value****: Indicates the significance of the effect and* ***η²_p_ (Eta squared partial):*** *Proportion of variance explained by each factor or interaction, relative to total variance (excluding error).*

|  | **Factor** | **SS** | **df** | **MS** | **F-statistic** | **p-value** | **η²_p_** |
| --- | --- | --- | --- | --- | --- | --- | --- |
| **SARA^home^**  **sum-score** | **Day** | 86 | 13 | 6.645 | 0.430 | 0.959 | ≤ 0.01 |
|  | **Time** | 1 | 1 | 0.874 | 0.057 | 0.812 | ≤ 0.01 |
|  | **Interaction** | 29 | 13 | 2.238 | 0.145 | 1.000 | ≤ 0.01 |
|  | **Residuals** | 17479 | 1132 | 15.441 |  |  |  |
|  | **Total** | 17595 | 1159 |  |  |  |  |
| ***Gait*** | ***Day*** | 9.7 | 13 | 0.748 | 0.347 | 0.984 | ≤ 0.01 |
|  | ***Time*** | 0 | 1 | 0.010 | 0.004 | 0.947 | ≤ 0.01 |
|  | ***Interaction*** | 5.3 | 13 | 0.410 | 0.190 | 0.999 | ≤ 0.01 |
|  | ***Residuals*** | 2899 | 1343 | 21.585 |  |  |  |
|  | ***Total*** | 2914 | 1370 |  |  |  |  |
| ***Stance*** | ***Day*** | 12.2 | 13 | 0.940 | 0.572 | 0.878 | ≤ 0.01 |
|  | ***Time*** | 0.9 | 1 | 0.922 | 0.561 | 0.454 | ≤ 0.01 |
|  | ***Interaction*** | 7.7 | 13 | 0.592 | 0.360 | 0.981 | ≤ 0.01 |
|  | ***Residuals*** | 2127.1 | 1294 | 16.438 |  |  |  |
|  | ***Total*** | 2147.9 | 1321 |  |  |  |  |
| ***Speech*** | ***Day*** | 8.2 | 13 | 0.631 | 0.455 | 0.949 | ≤ 0.01 |
|  | ***Time*** | 0.1 | 1 | 0.136 | 0.098 | 0.754 | ≤ 0.01 |
|  | ***Interaction*** | 4.8 | 13 | 0.366 | 0.264 | 0.996 | ≤ 0.01 |
|  | ***Total*** | 454.7 | 1354 |  |  |  |  |
| ***Nose-Finger*** | ***Day*** | 1.6 | 13 | 0.126 | 0.370 | 0.979 | ≤ 0.01 |
|  | ***Time*** | 0.5 | 1 | 0.477 | 1.403 | 0.236 | ≤ 0.01 |
|  | ***Interaction*** | 1.1 | 13 | 0.087 | 0.256 | 0.996 | ≤ 0.01 |
|  | ***Residuals*** | 451.5 | 1327 | 0.340 |  |  |  |
|  | ***Total*** | 454.7 | 1354 |  |  |  |  |
| ***Alternating Hands*** | ***Time*** | 0 | 1 | 0.020 | 0.033 | 0.857 | ≤ 0.01 |
|  | ***Interaction*** | 2.2 | 13 | 0.170 | 0.279 | 0.995 | ≤ 0.01 |
|  | ***Residuals*** | 810.8 | 1327 | 0.611 |  |  |  |
|  | ***Total*** | 815.7 | 1354 |  |  |  |  |

***Supplementary Table 4:*** *Results of* *Wilcoxon Rank-Sum Tests Comparing SARA^home^ score ranges between patients with (MoCA ≤25) and without (MoCA >25) cognitive impairments. Med = Median, IQR = Interquartile Range, p-values <0.05 are printed bold.*

| **Variables** | **MoCA<=25**  **Med (IQR)** | **MoCA>25**  **Med (IQR)** | **N_A_/N**_B_ | ***W statistic*** | ***p*-value** | ***Cohen’s r*** |
| --- | --- | --- | --- | --- | --- | --- |
| SARA^home^ (total) | 2.5 (2) | 3 (1.5) | 15/35 | 179 | 0.076 | 0.252 |
| Gait | 1 (1.25) | 1 (1) | 16/37 | 202 | **0.046** | 0.275 |
| Stance | 0.5 (2) | 1 (2) | 16/37 | 244 | 0.297 | 0.145 |
| Alternating Hands | 1 (0.625) | 1 (0.875) | 16/38 | 303 | 0.992 | 0.003 |
| Nose-Finger | 1 (0.5) | 0.5 (0.5) | 16/37 | 338 | 0.405 | 0.116 |
| Speech | 1 (0.5) | 1 (0) | 15/38 | 259 | 0.555 | 0.083 |

***Supplementary Table 5:*** *SARA^home^ and SARA scores at baseline (V1) and during a follow-up (V2) after a median interval of 411 days (IQR 366-547). SARA^home^ scores were calculated as means from four consecutive days of assessment. Results were compared with t-test.*

| **Score** | **V1 (Mean ± SD)** | **V2 (Mean ± SD)** | **T (df)** | **p-value** | **SRM** |
| --- | --- | --- | --- | --- | --- |
| **SARA^home^** | 6.87 ±3.22 | 8.08 ±2.92 | -2.22 (10) | 0.03 | 0.67 |
| **SARA** | 9.86 ±4.86 | 10.41 ±5.32 | -1.23 (10) | 0.25 | 0.37 |

***Supplementary Figure 1:*** *SARA^home^ assessment. SARA^home^ consists of five out of eight SARA items, including examination of gait and stance, speech disturbance, fast alternating hands movements, and the nose-finger test. Participants require a smart device with camera and 5m barrier-free walkway. The examination is video-recorded using an e-health app (ATOM5 by Aparito) for centralized rating.*


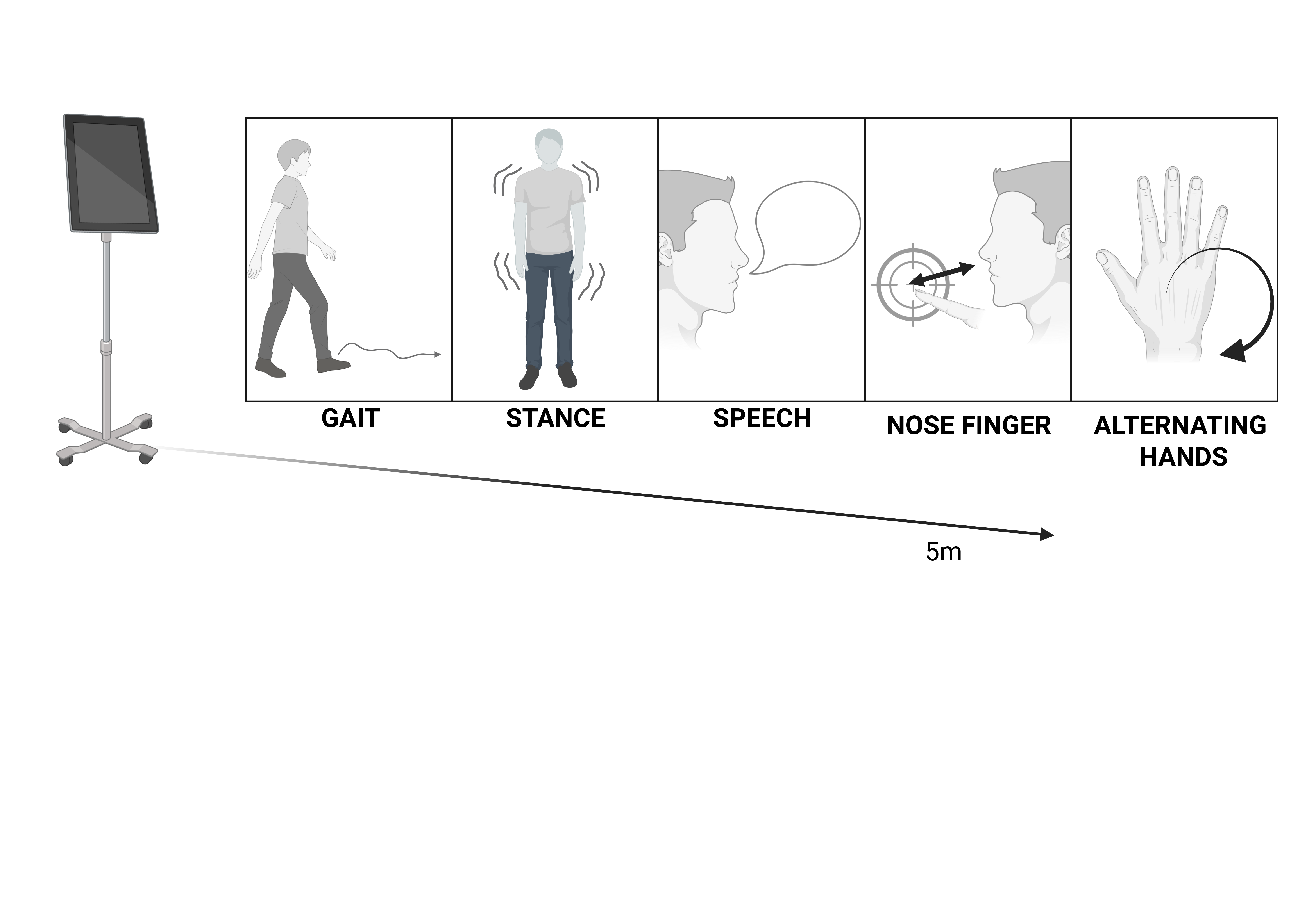


***Supplementary Figure 2:*** *Frequency distribution of variables SARA (a), CAG repeat length of the longer allele (b) and MoCa score (c) at baseline.*


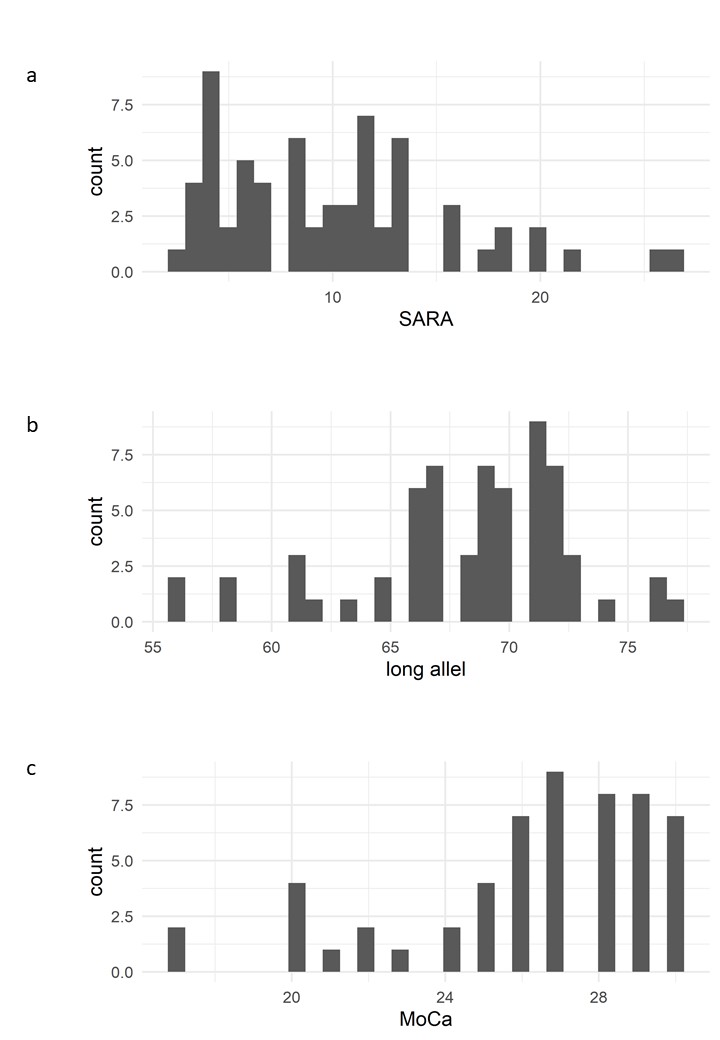


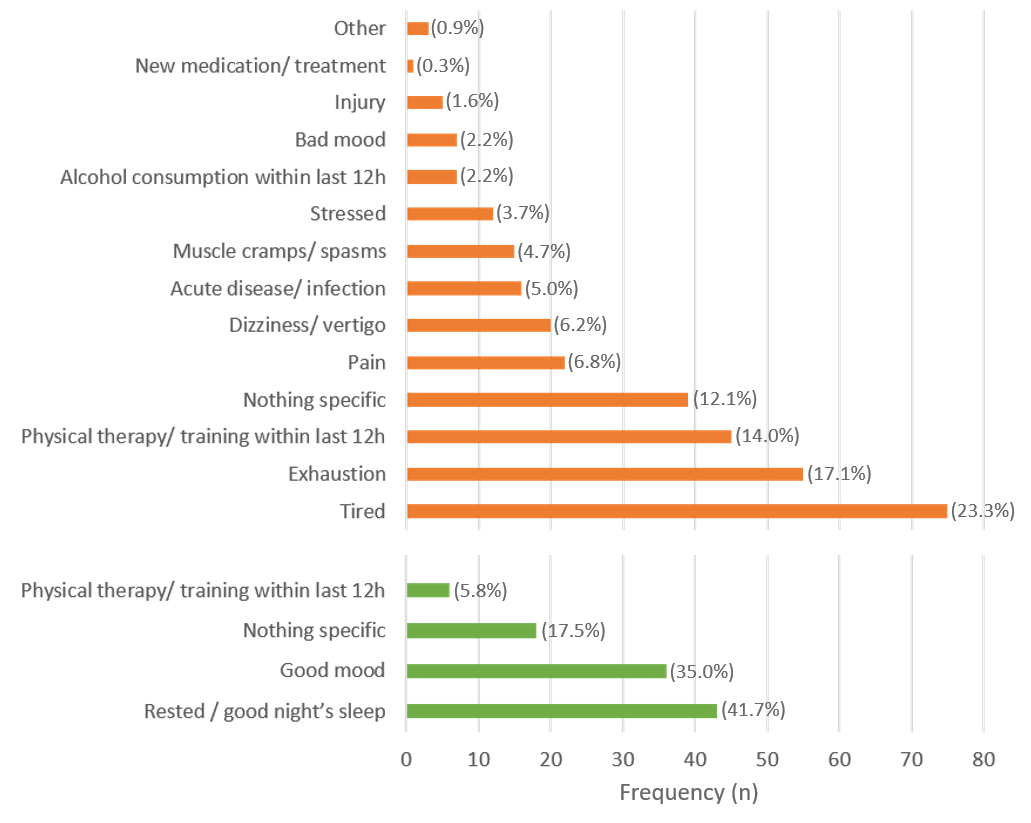
 ***Supplementary Figure 3:*** *Frequency list of reasons for changes in ataxia severity reported by patients. Red bars (top) show reported reasons for worsenings of ataxia severity (in 322 instances), green bars (bottom) show reasons for improvements (in 103 instances). Relative frequencies are provided in brackets behind the bars*

*.*
